# 超临界流体色谱‐紫外检测法测定利格列汀中的*S*-对映体

**DOI:** 10.3724/SP.J.1123.2025.05014

**Published:** 2026-06-08

**Authors:** Wanjie LI, Wei JIN, Qian LIU, Jun WANG, Jian LE

**Affiliations:** 1.复旦大学药学院，上海 201203; 1. School of Pharmacy，Fudan University，Shanghai 201203，China; 2.上海市药品检验研究院，上海 201203; 2. Shanghai Institute for Drug Control，Shanghai 201203，China; 3.国家药品监督管理局化学药品制剂质量分析重点实验室，上海 201203; 3. National Medical Products Administration Key Laboratory for Quality Analysis of Chemical Drug Preparations，Shanghai 201203，China

**Keywords:** 超临界流体色谱, 紫外检测, 利格列汀, *S-*对映体, 手性拆分, 质量控制, supercritical fluid chromatography （SFC）, ultraviolet detection, linagliptin, *S*-enantiomer, chiral separation, quality control

## Abstract

采用超临界流体色谱（SFC）‐紫外检测法建立了一种利格列汀及其*S-*对映体的拆分方法，对所建立的方法进行方法学验证，并应用于实际样品中*S-*对映体的检测。实验考察了*S-*对映体在6种色谱柱上的分离情况，对不同助溶剂进行了研究，并对柱温、背压、流速等色谱条件进行了优化。采用DAICEL CHIRALPAK AD-H（250 mm×4.6 mm，5 μm）色谱柱进行分离，以超临界CO_2_为流动相A，以乙醇-异丙醇（1∶1，体积比）溶液（含0.25%二乙胺和0.25%三氟乙酸）为流动相B，等度洗脱A-B（73∶27，体积比），流速1.5 mL/min，柱温为40 ℃，背压为15 MPa，进样量为6 μL，检测波长为220 nm，在此色谱条件下利格列汀与其*S-*对映体分离度为3.1，峰形良好；两者均在2～90 μg/mL范围内与峰面积呈良好的线性关系，相关系数分别为0.999 7和0.999 9（*n*=8），检出限均为0.8 μg/mL（*S/N*=3），定量限均为2 μg/mL（*S/N*=10）。原料药和片剂中*S*-对映体的平均加标回收率分别为97.4%（RSD=1.1%， *n*=9）和101.6%（RSD=1.2%， *n*=9）。3批原料和两家企业的3批制剂中均未检出*S*-对映体。本研究采用SFC方法分离利格列汀及其*S*-对映体，环保、灵敏，分离效率高，峰面积重复性好，不仅可为利格列汀的质量控制与药品质量标准中SFC方法的收载提供技术依据，也为其他手性药物的快速拆分与杂质控制提供了可借鉴的策略。

## Abstract (en)

A supercritical fluid chromatography （SFC） method coupled with UV detection was developed for the separation of linagliptin and its *S-*enantiomer. The method was validated and successfully applied to detect the *S-*enantiomer in real pharmaceutical samples. The separation of the enantiomer was investigated using six different chromatographic columns， and different cosolvents were studied. Chromatographic conditions， including column temperature， backpressure， and flow rate， were optimized. The DAICEL CHIRALPAK AD-H column （250 mm×4.6 mm， 5 μm） was used for separation. Supercritical CO_2_ served as mobile phase A， and ethanol-isopropanol （1∶1， volume ratio） containing 0.25% diethanolamine and 0.25% trifluoroacetic acid was used as mobile phase B. Isocratic elution was carried out at a ratio of A∶B=73∶27 （volume ratio） with a flow rate of 1.5 mL/min. The column temperature was set at 40 ℃， back pressure at 15 MPa， injection volume at 6 μL， and detection wavelength at 220 nm. Under these conditions， linagliptin and its *S-*enantiomer were separated with a resolution of 3.1 and good peak shapes. Both linagliptin and its *S*-enantiomer exhibited good linearity in the concentration range of 2–90 μg/mL， with correlation coefficients of 0.999　7 and 0.999　9 （*n*=8）， respectively. The limit of detection （LOD） for both was 0.8 μg/mL （*S/N*=3）， and the limit of quantification （LOQ） for both was 2 μg/mL （*S/N*=10）. The average recoveries of the *S*-enantiomer spiked at low， medium， and high concentrations in active pharmaceutical ingredients and tablets were 97.4% （RSD=1.1%， *n*=9） and 101.6% （RSD=1.2%， *n*=9）， respectively. *S*-Enantiomer was not detected in three batches of active pharmaceutical ingredients or in three batches of tablets from two different manufacturers. This study represents the first application of SFC for the separation of linagliptin and its *S*-enantiomer. The method is environmentally friendly， sensitive， and highly efficient， offering good repeatability of peak area. It provides a solid foundation for the quality control of linagliptin and the inclusion of the SFC method in pharmaceutical quality standards， while also offering a useful approach for the rapid separation and impurity control of other chiral drugs.

利格列汀（linagliptin）化学名为8-［（3*R*）-3-氨基-1-哌啶基］-7-（2-丁炔基）-3，7-二氢-3-甲基-1-［（4-甲基-2-喹唑啉基）甲基］-1*H*-嘌呤-2，6-二酮，是一种二肽基肽酶4（DPP-4）抑制剂，临床上用于帮助2型糖尿病患者控制血糖^［1］^。利格列汀通过与DPP-4结合，抑制其活性，从而延缓胰高血糖素依赖性胰岛素促进多肽（GIP）和胰高血糖素样肽-1（GLP-1）在体内的失活，使内源性GIP和GLP-1的水平升高，进而降低血糖水平^［2，3］^。利格列汀具有一个手性中心，存在一对对映体，结构式见图1。目前中国药典2020年版、美国药典2025年版、欧洲药典11版和英国药典2025年版均未收载利格列汀原料及制剂的质量标准。利格列汀临床使用的是*R*构型光学异构体，为了确保在合成和制剂过程中无*S*构型利格列汀产生，需要对其*S-*对映体进行控制^［4］^。因此，开发利格列汀对映体纯度的测定方法非常重要。目前已有报道采用HPLC^［5，6］^、毛细管电泳法（CE）^［4，7］^分离测定利格列汀及其*S-*对映体，但仍存在一些局限性，如保留时间较长、洗脱顺序不佳（杂质在主药之后洗脱）、使用大量烷烃类试剂（如正己烷）等。

**图1:**
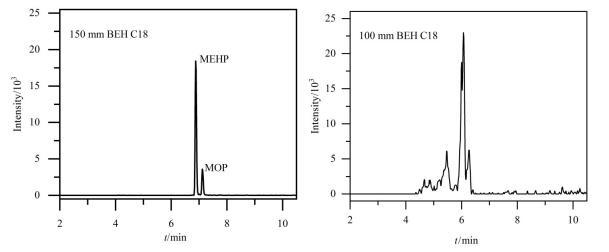
利格列汀及其 S-对映体的结构式

超临界流体色谱（SFC）是以超临界CO_2_为流动相的色谱方法，当物质处于高于其临界点的温度和压力时，会转变为超临界流体，这种流体展现出介于液体和气体之间的独特物理化学特性，兼具气相色谱的气体高扩散性和液相色谱的液体高溶解性，通常作为正相色谱技术^［8，9］^。其使用范围较广，任何可以溶解到甲醇或者极性更低的溶剂中的化合物都可以用SFC进行分析^［10］^。尤其是在分离对映异构体及其他异构体方面，SFC逐渐成为主流技术之一^［11-13］^。相比常规正相HPLC，SFC在分析速度和分离度方面都更加出色^［14，15］^。此外，对于极性较强的溶质，可以向流动相中加入有机改性剂（主要为低级醇类）改善洗脱强度和分离情况。对于强极性溶质，还可以向流动相中添加强极性添加剂（强酸或强碱）^［16］^，改善溶质与固定相过强的相互作用，改善洗脱时间和色谱峰峰形。

近年来，基于SFC原理的商业化仪器平台进一步发展为超高效合相色谱（UPC^2^）。此系统在硬件上借鉴超高效液相色谱（UPLC）的小体积管路与高压输送设计，但仍以超临界CO_2_为主要流动相，配合有机改性剂，并通过控制背压调节流动相密度与溶解能力，可视为SFC技术在现代仪器平台上的一种实现。

基于SFC在对映异构体分离方面的优势，以及超临界CO_2_的高扩散性和低黏度，本研究拟采用SFC开发一种利格列汀及其*S-*对映体的分离测定方法，并对利格列汀原料药及片剂中的*S-*对映体杂质进行检测，为其质量控制提供一种新的选择。

## 1 实验部分

### 1.1 仪器、材料和试药

Acquity UPC^2^超高效合相色谱系统（Waters公司，配有二极管阵列检测器）；XSE 205型天平（METTLER TOLEDO公司）； SK 2510HP型超声波清洗机（上海科导超声仪器有限公司）；Sigma 1-14离心机（Sigma Zentrifugen公司）。

二氧化碳（上海Air liquide公司）；甲醇（MeOH）、乙醇（EtOH）、异丙醇（IPA）、乙腈（ACN）、二乙胺（DEA）、三氟乙酸（TFA）均为色谱纯（国药集团化学试剂有限公司）。利格列汀对照品（批号：430047-202302，纯度：99.1%，中国食品药品检定研究院）、*S-*对映体对照品（批号：0221-RF-0065，纯度：99.6%，广州佳途科技股份有限公司）；利格列汀原料药（批号：021114、011114、011014，勃林格殷格翰公司）；利格列汀片（批号：2401045、2404048，扬子江药业集团有限公司；批号：AA7100A，勃林格殷格翰公司；规格均为5 mg）。

### 1.2 溶液的配制

系统适用性溶液：取适量利格列汀和*S-*对映体对照品，精密称定，加甲醇溶解并定量稀释成每1 mL中同时约含6 mg利格列汀和60 μg *S-*对映体的溶液。

消旋体溶液：取适量利格列汀和*S-*对映体对照品，精密称定，加甲醇溶解并定量稀释成每1 mL中同时约含利格列汀和*S-*对映体均为200 μg的溶液。

*S-*对映体对照品溶液储备液：精密称量适量的*S-*对映体对照品，加甲醇溶解，并定量稀释成约100 μg/mL的*S-*对映体对照品溶液。

供试品溶液：精密称取利格列汀原料药约30 mg，置于5 mL容量瓶中，加适量甲醇，超声使溶解，放至室温后加甲醇稀释至刻度，制成含利格列汀6 mg/mL的溶液，作为原料药供试品溶液；取利格列汀片适量，研细，精密称取细粉适量（每份约相当于利格列汀12 mg），置于2 mL容量瓶中，加适量甲醇，超声使溶解，放至室温后加甲醇稀释至刻度，摇匀，经10 000 r/min离心20 min后，取上清液，制成含利格列汀6 mg/mL的溶液，作为片剂供试品溶液。

### 1.3 色谱条件

色谱柱：DAICEL CHIRALPAK AD-H色谱柱（250 mm×4.6 mm，5 μm）；流动相：A为超临界CO_2_，B为乙醇-异丙醇（1∶1，体积比）溶液（含0.25%二乙胺和0.25%三氟乙酸）；等度洗脱：A-B（73∶27，体积比）；柱温：40 ℃；背压：15 MPa；流速：1.5 mL/min；检测波长：220 nm；进样量：6 μL。

## 2 结果与讨论

### 2.1 色谱柱的选择

多糖手性固定相广泛应用于手性化合物的对映体拆分，通过其手性选择剂（如纤维素或淀粉的衍生物）与手性分子之间的立体选择性相互作用实现分离。取含利格列汀和*S-*对映体均为100 μg/mL的溶液，按初始色谱条件进行分析。初始色谱条件如下：流动相为（A）超临界CO_2_-（B）0.1%二乙胺甲醇，等度洗脱A-B（75∶25，体积比），流速1.5 mL/min，柱温为40 ℃，背压为15 MPa，检测波长为220 nm，进样量为6 μL。对Welch Ultimate Amy-D （250 mm×4.6 mm， 5 μm）、DAICEL CHIRALCEL OD （250 mm×4.6 mm， 10 μm）、Waters Trefoil CEL1 （150 mm×3 mm， 2.5 μm）、Waters Trefoil AMY1 （150 mm×3 mm， 2.5 µm）、DAICEL CHIRALPAK AD （250 mm×4.6 mm， 10 μm）、DAICEL CHIRALPAK AD-H （250 mm×4.6 mm， 5 μm） 6种含有不同多糖衍生物填料、粒径大小和品牌的手性色谱柱进行考察，结果见图2。采用AMY1、Amy-D、OD、CEL1、AD色谱柱时均未能实现两种对映体的分离，最终选择AD-H进行进一步条件优化。

**图2:**
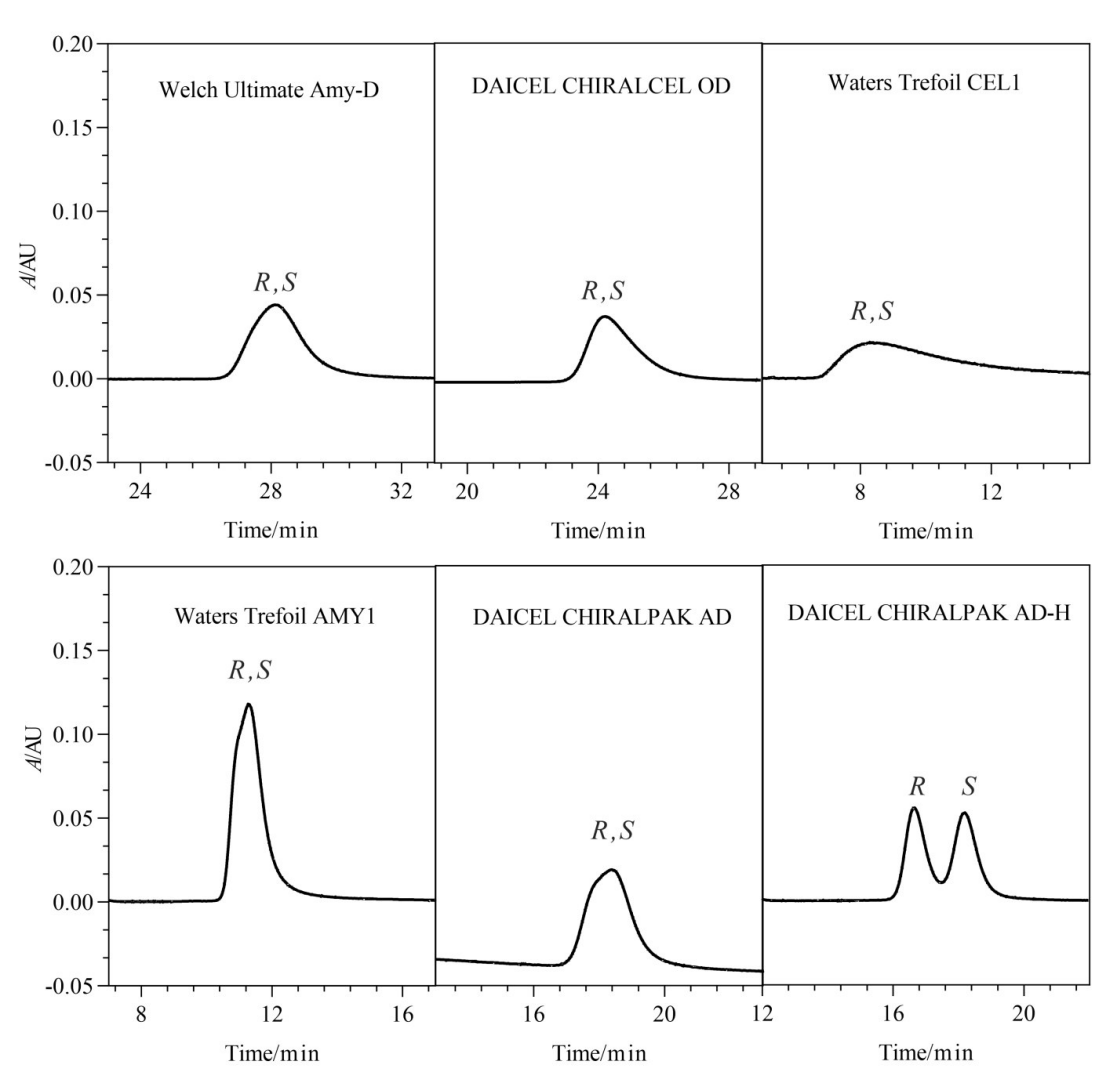
利格列汀及*S*-对映体在6种色谱柱上的色谱图

### 2.2 助溶剂的选择

超临界CO_2_虽然具有良好的溶解能力，但由于其介电常数较低，对极性溶质的溶解能力有限，流动相中改性剂和添加剂的比例会显著影响其对极性溶质的溶解和洗脱能力^［17］^。本实验考察了0.1%二乙胺甲醇溶液、甲醇-异丙醇（1∶1，体积比）溶液（含0.1%二乙胺和0.1%三氟乙酸）、甲醇-乙腈（1∶1，体积比）溶液（含0.1%二乙胺和0.1%三氟乙酸）、乙醇-乙腈（1∶1，体积比）溶液（含0.1%二乙胺和0.1%三氟乙酸）、甲醇-乙醇（1∶1，体积比）溶液（含0.1%二乙胺和0.1%三氟乙酸）、乙醇-异丙醇（1∶1，体积比）溶液（含0.1%二乙胺和0.1%三氟乙酸）等不同助溶剂对利格列汀及其*S*-对映体分离的影响，结果见图3。采用0.1%二乙胺甲醇溶液时，利格列汀及其*S-*对映体实现分离，但峰形较宽。采用乙醇-异丙醇（1∶1，体积比）溶液（含0.1%二乙胺和0.1%三氟乙酸）时利格列汀及其*S-*对映体分离度良好。考察采用乙醇-异丙醇（1∶1，体积比）溶液（含0.1%～0.25%二乙胺和0.1%～0.25%三氟乙酸）为助溶剂时，利格列汀及其*S*-对映体的分离情况。随着二乙胺和三氟乙酸加入比例的升高，利格列汀与*S*-对映体的分离度增加，响应值变高，保留时间缩短。综合考虑信噪比和理论塔板数情况，选择乙醇-异丙醇（1∶1，体积比）溶液（含0.25%二乙胺和0.25%三氟乙酸）为助溶剂。

**图3:**
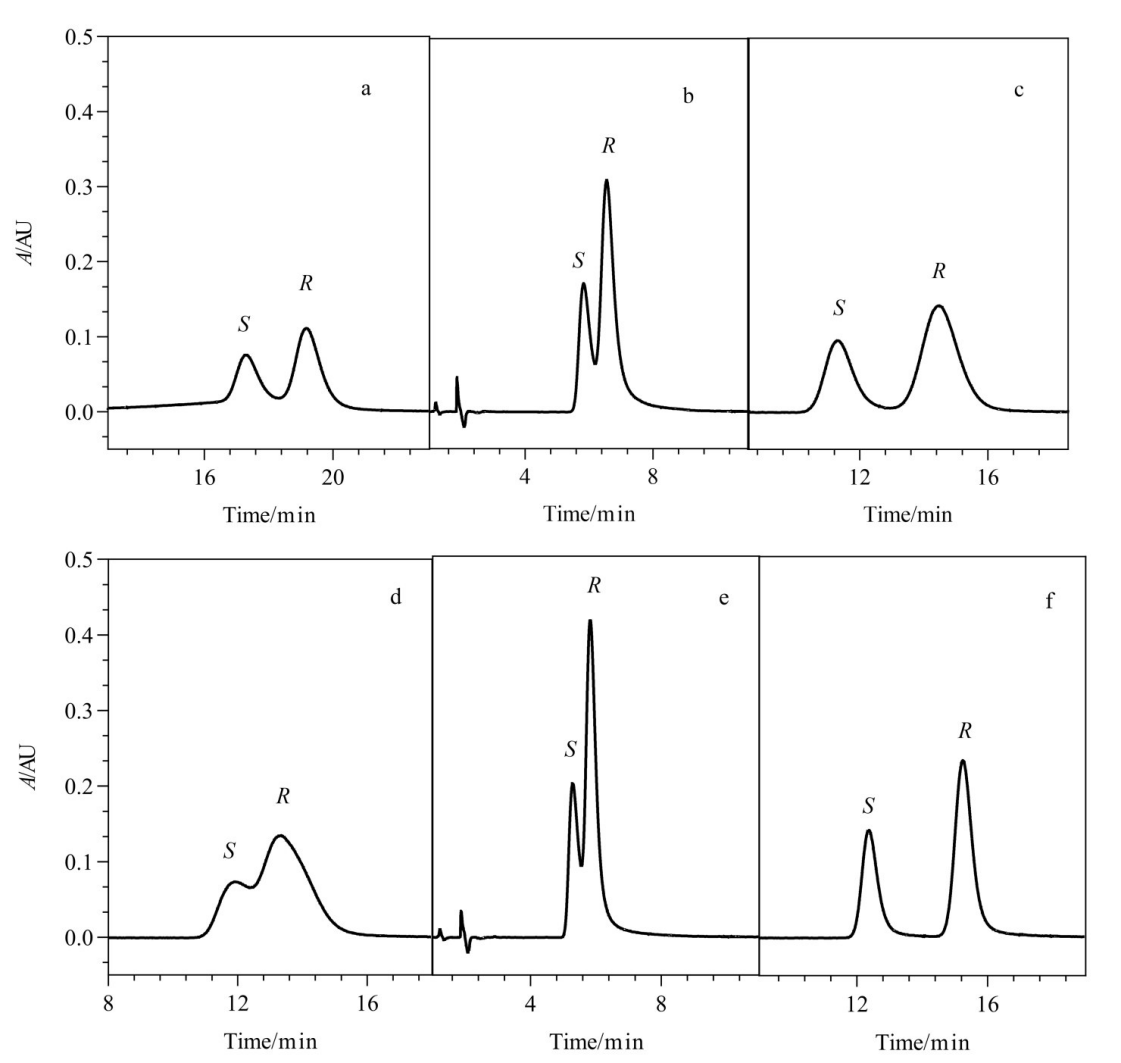
不同助溶剂对利格列汀及*S*-对映体分离效果的影响

### 2.3 流动相比例的选择

考察超临界CO_2_和乙醇-异丙醇（1∶1，体积比）溶液（含0.25%二乙胺和0.25%三氟乙酸）体积比（78∶22、75∶25、73∶27、72∶28、70∶30、65∶35）对利格列汀及其*S*-对映体分离的影响，结果见图4。随着超临界CO_2_体积比的降低，利格列汀及其*S*-对映体保留时间缩短，分离度降低，但体积比太高时峰形变差，峰展宽，灵敏度下降，综合考虑保留时间和分离度，选择超临界CO_2_-乙醇-异丙醇（1∶1，体积比）溶液（含0.25%二乙胺和0.25%三氟乙酸）体积比为73∶27。

**图4:**
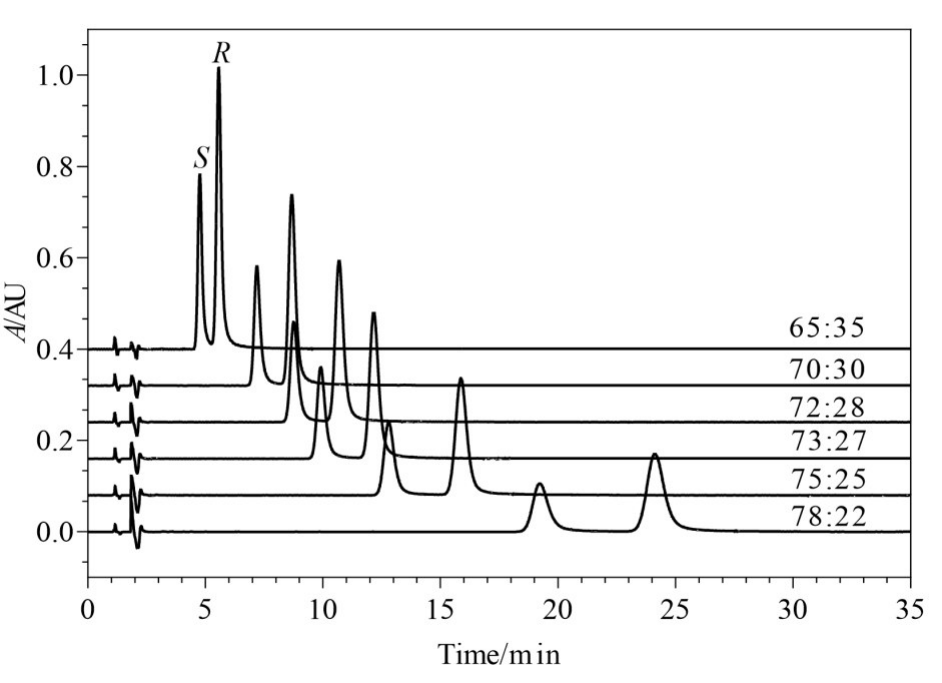
采用不同流动相比例时利格列汀及其*S-*对映体的色谱图

### 2.4 柱温的选择

超临界流体的密度随温度变化显著，密度的变化直接影响溶剂的溶解能力和溶质的保留行为^［18，19］^。研究考察了30～45 ℃柱温下利格列汀及其*S-*对映体的分离情况，结果见图5。随着柱温的升高，分离度增加，理论塔板数增加，综合考虑信噪比，选择40 ℃为优化柱温。

**图5:**
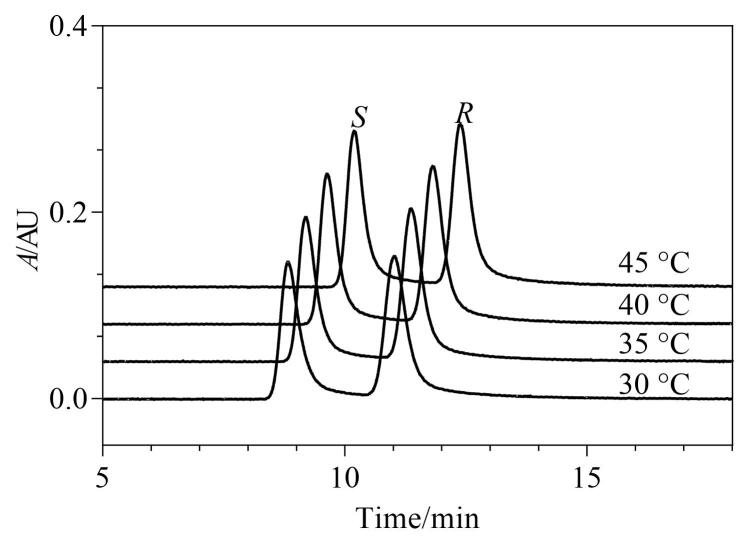
利格列汀及其*S*-对映体在不同柱温下的色谱图

### 2.5 背压的选择

增加背压通常会导致保留时间缩短，同时其变化可能会影响流动相的流动特性和界面分布，进而影响柱效^［20］^。研究考察了11～20 MPa背压下利格列汀及其*S-*对映体的分离情况。随着背压的升高，流动相的洗脱能力增强，分离度降低，信噪比降低，结果见图6，在13 MPa和15 MPa下都可以得到较好的分离效果。综合考虑保留时间和分离度、信噪比，选择15 MPa为优化背压。

**图6:**
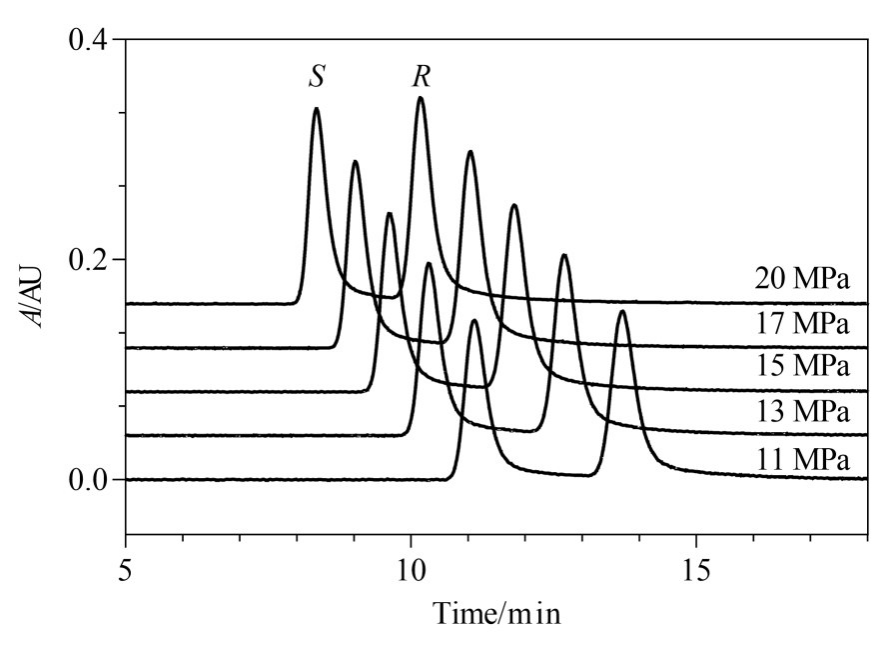
不同背压对利格列汀及其*S*-对映体分离效果的影响

### 2.6 流速的选择

在SFC中，流动相流速是一个关键的操作参数，流速增加，传质效率通常会提高。研究考察了1.0、1.5、2.0、3.0 mL/min流速下利格列汀及其*S-*对映体的分离情况，结果见图7。随着流速增加，分离度降低，综合考虑保留时间和分离度，选择1.5 mL/min为最终流速。

**图7:**
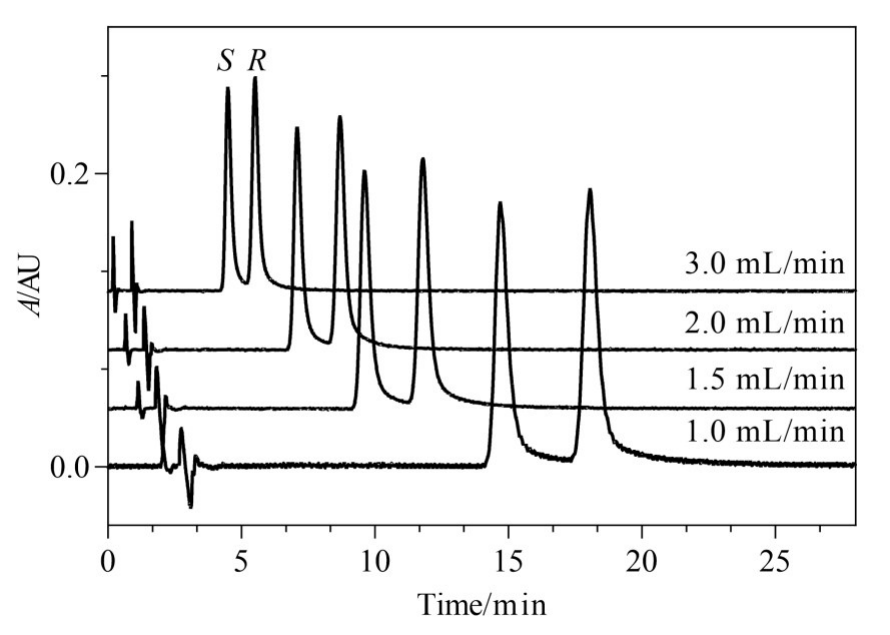
不同流速对利格列汀及其*S-*对映体分离效果的影响

### 2.7 方法学考察

#### 2.7.1 线性范围和灵敏度

分别精密量取利格列汀对照品，加入适量*S-*对映体对照品储备液，用甲醇定量稀释成同时含利格列汀和*S-*对映体均为2.0、4.5、9.0、18.0、36.0、60.0、72.0、90.0 μg/mL的系列溶液。按1.3节色谱条件进样，记录色谱图，以利格列汀和*S-*对映体的质量浓度（*X*）为横坐标，峰面积（*Y*）为纵坐标，进行线性回归，得到利格列汀和*S-*对映体的线性回归方程：*Y*=24 910*X*+734.72，相关系数*r*^2^=0.999 7（*n*=8）；*Y*=25 057*X*+857.24，*r*^2^=0.999 9（*n*=8）。两者的峰面积均与进样浓度呈良好的线性关系。按*S/N*分别为3和10计算检出限和定量限，利格列汀及其*S-*对映体检出限均为0.8 μg/mL，定量限均为2 μg/mL。

#### 2.7.2 日内和日间精密度

按1.3节色谱条件，对含*S*-对映体分别为18.0、36.0、60.0 μg/mL的低、中、高 3个水平的标准溶液分别进行日内（24 h）和日间（72 h）精密度考察。日内（24 h）*S-*对映体峰面积的RSD分别为1.38%、1.20%、0.68%；日间（72 h）*S-*对映体峰面积的RSD分别为3.37%、2.78%、2.71%。

#### 2.7.3 加标回收率试验

精密称取一批利格列汀原料药（批号：021114）9份，每份30 mg，分别置于5 mL量瓶中，分别精密加入*S-*对映体对照品储备液1.0、1.5、3.0 mL，每个水平各3份，加适量甲醇，摇匀，加甲醇稀释至刻度，制成含*S-*对映体低、中、高3个不同水平的溶液。按1.3节色谱条件进样，按标准曲线法计算每份溶液的加标回收率。低、中、高3个不同水平下*S*-对映体的平均回收率分别为97.61%、97.83%和96.88%，总的平均回收率为97.4%（*n*=9），RSD分别为1.43%、1.25%和0.69%，平均RSD为1.12%（*n*=9）。

取一批利格列汀片（批号：2401045），研细，精密称取细粉9份，每份约相当于利格列汀12 mg，置于2 mL量瓶中，分别加入*S-*对映体对照品储备液0.4、0.6、1.2 mL，每个水平各3份，超声使溶解，放至室温，加甲醇稀释至刻度，离心后取上清液，制成含*S-*对映体低、中、高3个不同水平的溶液。按1.3节色谱条件，采用标准曲线法计算每份溶液的加标回收率。低、中、高3个不同水平下*S*-对映体的平均回收率为103.58%、100.07%和101.12%，总的平均回收率为101.6%（*n*=9），RSD分别为1.80%、1.34%和0.56%，平均RSD为1.23%（*n*=9）。

### 2.8 供试品溶液稳定性考察

取加标回收率试验中含*S-*对映体质量浓度为60 μg/mL的加标溶液，在15 ℃下连续放置12 h，分别于2、5、8、10、12 h各进样6 μL，考察溶液的稳定性。供试品溶液在0～12 h内各时间点的峰面积与0 h峰面积的百分比均在99%～102%范围内，*S-*对映体峰面积的RSD（*n*=6）为0.91%，表明供试品溶液在12 h内稳定。

### 2.9 方法应用

取系统适用性溶液及原料药和片剂的供试品溶液，按1.3节色谱条件分别进样，色谱图见图8，系统适用性溶液中利格列汀与其*S-*对映体保留时间适宜，分离度为3.1。供试品溶液中均未检出*S-*对映体，说明原料拆分过程纯化完全，制剂无消旋化产生，生产工艺稳定。

**图8:**
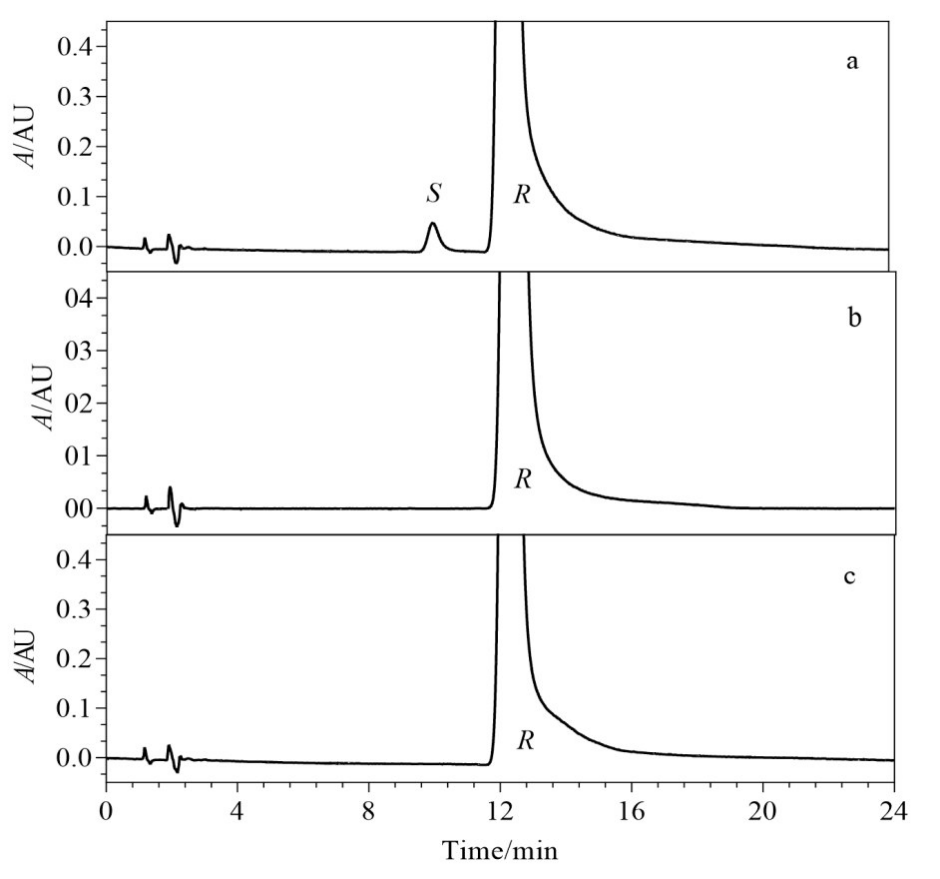
利格列汀（a）系统适用性溶液、（b）原料药（批号：021114）和（c）片剂（批号：AA7100A）的典型色谱图

## 3 结论

本研究考察了多糖手性色谱柱、流动相、背压、柱温等对利格列汀及其*S*-对映体分离的影响，最终采用硅胶表面涂敷直链淀粉-三（3，5-二甲基苯基氨基甲酸酯）（DAICEL CHIRALPAK AD-H）色谱柱建立了SFC拆分方法，实现了利格列汀与*S*-对映体的基线分离，且*S*-对映体先于主药出峰，有利于杂质控制。然而，流动相中二乙胺-三氟乙酸的协同机制尚未阐明。相较于传统正相HPLC方法，本研究所建立的SFC方法在分离效率、环保性与分析速度方面均表现出明显优势，避免了大量烷烃类有机溶剂的使用，符合绿色分析化学的发展趋势。该方法不仅可为利格列汀的质量控制与药品质量标准中SFC方法的收载提供技术依据，也为其他手性药物的快速拆分与杂质控制提供了可借鉴的策略。
